# Supplementary material for: The Influence of Transcription Factor Competition on the Relationship between Occupancy and Affinity
Source: PLoS One. 2013 Sep 27;8(9):e73714. doi: 10.1371/journal.pone.0073714 (PMC3785477; doi:10.1371/journal.pone.0073714)
Supplement: File S1 — This file contains Table S1 and Table S2. Table S1, TF species default parameters. Table S2. lacI PWM. (PDF) [file pone.0073714.s010.pdf]

Supplementary Material to:
The influence of TF competition on the relationship between occupancy and affinity

Nicolae Radu Zabet[1,2,*], Robert Foy[1,2], Boris Adryan[1,2,†]

1 Cambridge Systems Biology Centre, University of Cambridge, Tennis Court Road, Cambridge CB2 1QR, UK

2 Department of Genetics, University of Cambridge, Downing Street, Cambridge CB2 3EH, UK

∗ **E-mail:** n.r.zabet@gen.cam.ac.uk

† **E-mail:** ba255@cam.ac.uk

Table S1. TF species default parameters

parameter	lacI	non-cognate	notation
copy number	see main manuscript		TF_x
motif sequence	see Table S2	-	
energetic penalty for mismatch	1 K_BT	13 K_BT	ε_x^*
nucleotides covered on left	0 bp	23 bp	TF_x^{left}
nucleotides covered on right	0 bp	23 bp	TF_x^{right}
association rate to the DNA	see main manuscript		k_x^{assoc}
unbinding probability	0.001474111	0.001474111	P_x^{unbind}
probability to slide left	0.4992629	0.4992629	P_x^{left}
probability to slide right	0.4992629	0.4992629	P_x^{right}
probability to dissociate completely when unbinding	0.1675	0.1675	P_x^{jump}
time bound at the target site	$1.18E-6\ s$	$0.3314193\ s$	τ_x^0
the size of a step to left	1 bp	1 bp	
the size of a step to right	1 bp	1 bp	
variance of repositioning distance after a hop	1 bp	1 bp	σ_{hop}^2
the distance over which a hop becomes a jump	100 bp	100 bp	d_{jump}
the proportion of prebound molecules	0.0	0.9	
affinity landscape roughness	-	1.0 K_BT	

Table S2. lacI PWM

**Position**	A	C	G	T
1	0.6200	−0.6900	0.1400	−0.6900
2	0.6200	−0.6900	0.1400	−0.6900
3	0.1600	0.1400	−0.6900	0.1800
4	0.1600	−0.6900	−0.6900	0.6200
5	−0.7000	−0.7000	0.9000	−0.7000
6	−0.6900	−0.6900	−0.6900	0.9300
7	0.0077	−0.0084	−0.0073	0.0083
8	0.0077	−0.0084	−0.0073	0.0083
9	0.0077	−0.0084	−0.0073	0.0083
10	0.0077	−0.0084	−0.0073	0.0083
11	0.0077	−0.0084	−0.0073	0.0083
12	0.0077	−0.0084	−0.0073	0.0083
13	0.0077	−0.0084	−0.0073	0.0083
14	0.0077	−0.0084	−0.0073	0.0083
15	0.0077	−0.0084	−0.0073	0.0083
16	0.6200	−0.6900	0.1400	−0.6900
17	−0.7000	0.9000	−0.7000	−0.7000
18	0.9300	−0.6900	−0.6900	−0.6900
19	0.9300	−0.6900	−0.6900	−0.6900
20	−0.6900	0.1400	−0.6900	0.6200
21	−0.6900	0.1400	−0.6900	0.6200

Generating the *in silico* ChIP profile

```
generateChIPProfile <- function(input.vec, mean, sd, smooth = NULL) {
    var = sd^2
    shp = mean^2/var
    scl = var/mean
    l = length(input.vec)

    f = dgamma(0:length(input.vec), shape = shp, scale = scl)
    F = rev(cumsum(rev(f)))

    peak.centres = which(input.vec > mean(input.vec))
    peaks = vector("numeric", l)

    for(pc in peak.centres) {
        this.peak = vector("numeric", l)
        this.peak[pc:l] = F[1:(l-pc+1)]
        this.peak[1:(pc-1)] = F[pc:2]
        peaks = peaks + this.peak * input.vec[pc]
    }

    if(!is.null(smooth)){
        if((smooth %% 2) == 0){smooth = smooth - 1}
        mid = round(smooth/2,0) + 1
        d = smooth - mid
        for(i in mid:(length(peaks) - d)) {
            peaks[i] = mean(peaks[max(0,(i-d)):min(length(input.vec),(i+d))])
        }
    }

    return(peaks)
}
```
